# Intensive training induces longitudinal changes in meditation state-related EEG oscillatory activity

**DOI:** 10.3389/fnhum.2012.00256

**Published:** 2012-09-10

**Authors:** Manish Saggar, Brandon G. King, Anthony P. Zanesco, Katherine A. MacLean, Stephen R. Aichele, Tonya L. Jacobs, David A. Bridwell, Phillip R. Shaver, Erika L. Rosenberg, Baljinder K. Sahdra, Emilio Ferrer, Akaysha C. Tang, George R. Mangun, B. Alan Wallace, Risto Miikkulainen, Clifford D. Saron

**Affiliations:** ^1^Department of Psychiatry and Behavioral Sciences, Stanford UniversityStanford, CA, USA; ^2^Department of Computer Science, University of Texas at AustinTX, USA; ^3^Department of Psychology, University of CaliforniaDavis, CA, USA; ^4^Center for Mind and Brain, University of CaliforniaDavis, CA, USA; ^5^Department of Psychiatry and Behavioral Sciences, Johns Hopkins UniversityBaltimore, MD, USA; ^6^Mind Research NetworkAlbuquerque, NM, USA; ^7^School of Social Sciences and Psychology, University of Western SydneySydney, NSW, Australia; ^8^Department of Psychology, University of New MexicoAlbuquerque, NM, USA; ^9^Department of Neurology, University of CaliforniaDavis, CA, USA; ^10^Santa Barbara Institute for Consciousness StudiesSanta Barbara, CA, USA; ^11^The M.I.N.D. Institute, University of CaliforniaDavis, Sacramento, CA, USA

**Keywords:** training, attention, meditation, beta, individual alpha frequency, EEG

## Abstract

The capacity to focus one's attention for an extended period of time can be increased through training in contemplative practices. However, the cognitive processes engaged during meditation that support trait changes in cognition are not well characterized. We conducted a longitudinal wait-list controlled study of intensive meditation training. Retreat participants practiced focused attention (FA) meditation techniques for three months during an initial retreat. Wait-list participants later undertook formally identical training during a second retreat. Dense-array scalp-recorded electroencephalogram (EEG) data were collected during 6 min of mindfulness of breathing meditation at three assessment points during each retreat. Second-order blind source separation, along with a novel semi-automatic artifact removal tool (SMART), was used for data preprocessing. We observed replicable reductions in meditative state-related beta-band power bilaterally over anteriocentral and posterior scalp regions. In addition, individual alpha frequency (IAF) decreased across both retreats and in direct relation to the amount of meditative practice. These findings provide evidence for replicable longitudinal changes in brain oscillatory activity during meditation and increase our understanding of the cortical processes engaged during meditation that may support long-term improvements in cognition.

The successful execution of adaptive, goal-directed behavior depends critically on the ability to focus and sustain attention. Individuals vary considerably in their ability to flexibly manage attention in accord with changing situational demands, and even brief lapses of attention can substantially disrupt performance of routine daily tasks (Padilla et al., 2006; Weissman et al., 2006). Moreover, chronic or long-term deficits in attentional control can significantly impair social and emotional functioning by contributing to dysfunctional patterns of affect regulation (Wadlinger and Isaacowitz, 2011) and inhibitory self-control (Sarter and Paolone, 2011). The potential salutary effects of stable attention on mental well-being have long been acknowledged by contemplative traditions, which have developed various mental training techniques thought to increase attentional capacity. However, despite accumulating evidence for the behavioral effects of such training (Hölzel et al., 2011b; Slagter et al., 2011), it is relatively unknown how the engagement of specific cognitive processes during meditative practice may support trait-like improvements in cognition and well-being.

Within many Buddhist contemplative traditions, the term *meditation* connotes a process of familiarizing (Tibetan: *gom*) oneself with subjective mental experience and cultivating (Sanskrit: *bhavana*) balanced qualities of mind (Wallace, 2005). Meditative training includes methods for developing enduring psychological traits through deliberate application of awareness to the contents of subjective experience, including thoughts, sensations, intentions, and emotions. Repeated practice of attending to one's experience is thought to cultivate familiarity with and insight into transient phenomenological experiences, leading to lasting changes in cognitive and psychological functioning (Ekman et al., 2005; Wallace and Shapiro, 2006). Among the practices derived from the Buddhist tradition is a class of attention-regulatory techniques designed to foster attentional stability and vividness, traditionally known as *shamatha* (*calm abiding*; Wallace, 2005, 2006). During shamatha practice, attention is directed to and sustained on an external or internal object of focus (e.g., sensations of the breath). While attention is focused on the meditative object, one continuously monitors the quality of attention and remains vigilant for distracting thoughts and impulses. When concentration inevitably lapses, attention is gently returned to the initial object of focus. These traditional descriptions of the mental processes employed during shamatha and other focused attention (FA) meditation techniques share considerable theoretical overlap with contemporary cognitive and neuroscientific theories of attention (Lutz et al., 2008; Slagter et al., 2011).

Recent longitudinal studies of intensive FA meditation have demonstrated training-related improvements in attentional stability (Lutz et al., 2009; MacLean et al., 2010) and alerting (Jha et al., 2007), sustained response inhibition (Sahdra et al., 2011), and information processing efficiency (van Vugt and Jha, 2011). In the same cohort of participants detailed in the current study, MacLean et al. (2010) observed behavioral improvements in perceptual discrimination and sustained attention during a 32-min visual discrimination task following three months of FA meditation. Additionally, there is evidence that these general behavioral improvements may be accompanied by functional and structural changes in cortical regions associated with attention and sensory processing. In a cross-sectional study of meditative adepts, Brefczynski-Lewis et al. (2007) reported increased activity in a broad network of attention-related brain regions, including frontoparietal, temporal, and posterior occipital cortical areas during meditation. Meditation experience has also been linked to increased cortical thickness in areas associated with attention and sensory processing, including regions within frontal and somatosensory cortex (Lazar et al., 2005; Grant et al., 2010; Hölzel et al., 2011a).

Taken together, the available evidence suggests that FA training leads to increased recruitment of attention-related brain networks during practice. Furthermore, recruitment and training of these attentional processes during meditation appears to generalize to improvements in the ability to focus and sustain attention across a wide variety of novel tasks. Slagter et al. (2011) characterized this kind of generalizable training as *process-specific learning* (cf. task-specific learning; Green and Bavelier, 2008). However, the nature of the sensory and cognitive processes invoked and trained during FA meditation is not yet well characterized, and relatively little is known about how repeated engagement of these processes may lead to long-term, trait-level improvements. Given that these potential improvements likely reflect training-induced changes in practice-associated brain networks, a detailed characterization of patterns of cortical activation during meditation is critical to understanding the role of meditative states in trait-level improvements. One avenue for inferring process-specific training of attention is by examining the modulation of task-specific cortical oscillations during meditation (Cahn and Polich, 2006).

Intrinsic rhythmicity in ongoing electrical cortical activity is traditionally organized into standard spectral frequency bands, ranging from slow- to fast-wave oscillations (Steriade, 2006). It is well-established that patterns of ongoing oscillatory activity across these frequency bands reflect functional state changes in the brain. For example, specific mental states such as wakefulness (e.g., drowsy vs. alert) and attention (e.g., distracted vs. focused) are known to be associated with the pattern of ongoing oscillatory activity in predictable ways. Oscillatory activity has also been linked to local- and large-scale synchronization of neuronal assemblies across brain regions (Varela et al., 2001; Fries, 2005; Siegel et al., 2012; Tallon-Baudry, 2012), which may facilitate processes dependent on the integration of information across distributed brain networks. There is increasing evidence that attentional modulation of neuronal oscillations may serve to influence selective sensory processing (Womelsdorf and Fries, 2007). Thus, patterns of oscillatory activity may be used to infer activation of process-specific cognitive processes due to task-specific attentional modulation during meditation.

Shamatha techniques such as *mindfulness of breathing* require repeated spatial and temporal orienting of attention to subtle tactile sensations and perceptions. Attentional modulations of ongoing oscillations in the alpha- (~8–12 Hz) and beta-bands (~13–30 Hz) have been functionally implicated in the perceptual processing of somatosensory information and may therefore serve as potential physiological markers of the capacity to focus attention on the breath. Activity in the alpha- and beta-bands is inversely related to cortical excitability (Tamura et al., 2005; Ploner et al., 2006; Ritter et al., 2009), speed of visual and sensorimotor processing (Thut et al., 2006; van Ede et al., 2011), stimulus discriminability (van Dijk et al., 2008), target detection accuracy (Linkenkaer-Hansen et al., 2004; Romei et al., 2010), and attentional suppression of distracting information (Snyder and Foxe, 2010; Haegens et al., 2012). Observed reductions in oscillatory activity in these bands may reflect the desynchronization of underlying neuronal populations (Pfurtscheller and Lopes da Silva, 1999), leading to an enhanced signal-to-noise ratio in preparation for upcoming sensory processing. Beta-band activity, for example, exhibits spatially and temporally specific modulations in anticipation of tactile stimulation. Attentional orienting to upcoming tactile stimuli induces hemisphere-specific suppression of beta oscillations in sensorimotor cortex (Dockstader et al., 2010; Jones et al., 2010; van Ede et al., 2010, 2011), suggesting a functional role for beta in spatially oriented attention. Prestimulus beta reductions over sensorimotor regions are also associated with enhanced conscious perception of subtle tactile stimuli (Linkenkaer-Hansen et al., 2004; Schubert et al., 2009). Schubert et al. (2009), for example, reported that individuals with lower absolute magnitudes of prestimulus beta synchronization showed reduced interference from perceptual masking while detecting tactile stimuli.

Reductions in alpha oscillations over sensorimotor cortex have also been linked to sensory detection and attentional orienting to tactile stimuli (Linkenkaer-Hansen et al., 2004; Jones et al., 2010; Haegens et al., 2012). Although a number of studies have reported modulations of ongoing oscillatory activity in the alpha-band during meditative states, alpha activity may partly reflect non-specific effects related to general arousal or to experimental task order (Cahn et al., 2010). Furthermore, because a wide array of contemplative practices and techniques have been linked to changes in alpha activity, the potential functional significance of alpha-band activity during FA meditation remains unclear (Cahn and Polich, 2006). Kerr et al. (2011) have provided initial support for the hypothesis that meditation training produces changes in cortical activation during attentional orienting to task-relevant stimuli. Compared to a control group, participants who completed a non-intensive eight-week course in mindfulness meditation demonstrated greater alpha-band suppression in response to anticipatory cues requiring the spatial orienting of attention to anticipated tactile stimuli. Notably, however, no evidence was found for changes in beta-band oscillations.

There are a number of methodological considerations that limit the conclusions that can be drawn from the existing literature. First, outcomes from most previous studies were based on cross-sectional comparisons of self-selected participants (i.e., experienced meditators) with only demographically matched controls, making it difficult to draw conclusions about the causal role of meditative practice in any reported differences in cortical activation patterns. The use of longitudinal designs and more rigorous control conditions are clearly necessary to rule out factors unrelated to meditation training (Jensen et al., 2012). Second, there are methodological limitations in the spectral analysis of ongoing cortical activity. Power spectrum estimates are susceptible to contamination by non-cortical sources of noise (e.g., muscle and ocular artifacts). In addition, the designation of frequency bands may not be sensitive to intra- and inter-individual differences that may influence the distribution of spectral frequencies. The understanding of cortical oscillatory activity during meditation may benefit from methodological advances in signal processing and the use of individualized frequency bands for the classification of spectral power (e.g., Klimesch, 1999).

In the present study, we addressed these issues by conducting a longitudinal wait-list controlled study of intensive meditation training using methodological advances in signal processing. Participants were randomly assigned to either an initial training group or a wait-list control group. Participants in the initial training group engaged in a three-month residential retreat wherein they received instruction and training in FA meditation (shamatha). During this period, participants assigned to the wait-list control condition served as a non-training comparison group. Subsequently, the wait-list control participants received formally identical training in a separate three-month residential retreat. To characterize training-related changes in cortical activity during meditation (i.e., state-dependent changes), we examined ongoing oscillatory activity while participants engaged in 6 min of mindfulness of breathing. Ongoing cortical oscillatory activity was assessed using spectral analysis of dense-array scalp-recorded electroencephalogram (EEG) data at three assessment points during each retreat. We used second-order blind source separation (SOBI; Belouchrani et al., 1997), along with a novel artifact removal tool (Saggar, 2011), to identify and remove signal sources of putative non-neural origin. Each participant's individual alpha frequency (IAF; Klimesch, 1999) was used to define spectral frequency bands.

We hypothesized that intensive training in FA meditation would promote increased recruitment of cortical regions associated with tactile sensory processing during meditation, because the meditation training specifically involved focusing attention on the breath and oral-nasal facial regions. Specifically, we predicted training-related changes in areas involved in attention and somatosensory processing as evidenced by reductions in alpha- and beta-band power across central and parietal areas of the scalp. We also investigated activity across the remaining spectral frequency bands based on previous reports of meditation-state-dependent activity in the delta, theta (Cahn and Polich, 2006), and gamma bands (Lutz et al., 2004; Cahn et al., 2010). Additionally, we predicted that intensive FA practice would lead to training-related changes in IAF during meditation. Because several meditation studies have revealed an overall slowing in oscillations within the alpha frequency, both as a trait and as a state effect (Cahn and Polich, 2006), we predicted a similar downward shift in IAF following training. Finally, we predicted that increases in cortical activity (reductions in beta- and alpha-band power) and decreases in IAF would vary in direct relation to the amount of time individuals spent engaging in FA meditation during the retreat.

## Materials and methods

### Participants

Participants were recruited through advertisements in various Buddhist magazines and email lists. Sixty participants were selected (out of 142 applicants) based on age, availability, physical and mental health, and previous retreat experience. These participants were then assigned to either an initial retreat (*N* = 30) or wait-list control (*N* = 30) group through stratified matched assignment. Groups were matched on age, sex, meditation experience, and ethnicity (see MacLean et al., 2010; Sahdra et al., 2011, for full assignment and matching criteria). During the initial retreat (Retreat 1), retreat group participants underwent three months of training at a remote mountain meditation center (Shambhala Mountain Center, Red Feather Lakes, CO). Participants lived and practiced onsite for the duration of training. Wait-list control group participants were flown to the retreat center for testing at each assessment point during Retreat 1 (data were collected after acclimatization for 72–96 h). Approximately three months after completion of Retreat 1, these same wait-list control group participants underwent formally identical training in a second three-month retreat (Retreat 2). The experimental staff was not blind to group membership. All study procedures were approved by the institutional review board of the University of California, Davis. Participants gave informed consent and were debriefed following training. Training participants paid for their room and board during each retreat (~$5300) but were compensated $20 for each hour of data collection.

EEG data from 22 participants in each group (retreat and wait-list control) were included in the analysis. Participants were excluded from analysis if their data at any single assessment point were not usable (due to very poor EEG signal quality, missing experimental event timing, or electrode location information). Initial retreat and wait-list control participants included in the analyses did not differ (all *p*s > 0.05) in age (retreat: *M* = 49.5, SD = 13.5, control: *M* = 44.2, SD = 15.8), gender (retreat: 12 female, control: 11 female), or lifetime meditation experience (retreat: *M* = 2855.6 h, SD = 2994.1, control: *M* = 2272.7 h, SD = 2326.3).

### Training

Participants practiced meditation under the guidance of Dr. B. Alan Wallace, an established Buddhist teacher, contemplative, and scholar. Meditative training included two general classes of techniques drawn from the Buddhist contemplative tradition: shamatha techniques that involve sustaining attention on a chosen object and ancillary techniques aimed at generating benevolent aspirations for the well-being of self and others (Wallace, 2006; Sahdra et al., 2011). Shamatha techniques included *mindfulness of breathing*, in which attention is directed toward the breath; *observing mental events*, in which attention is directed toward the whole field of mental experience (thoughts, images, sensations); and *observing the nature of consciousness*, in which attention is directed toward the experience of being aware. Beneficial aspirations included practices that cultivated *loving-kindness*, *compassion*, *empathic joy*, and *equanimity* (Wallace, 2006). Participants who received training in the first retreat (*M* = 5.7 h per day, SD = 1.5) or the second retreat (*M* = 5.4 h per day, SD = 1.5) spent a similar amount of time practicing solitary FA meditation [*t*_(42)_ = −0.486, *p* = 0.629]. In addition to engaging in solitary practices, participants met twice daily for group meditation practice and discussion guided by Dr. Wallace. Participants also met with Dr. Wallace privately once a week for individual advice, clarification, and guidance. Dr. Wallace was not present during any data collection procedures.

### Data collection

Participants were assessed at three points over the course of each retreat—at the beginning (pre), in the middle (mid), and at the end (post). At each assessment point, participants completed a battery of cognitive and affective measures over a two-day testing period (reported elsewhere, e.g., MacLean et al., 2010; Sahdra et al., 2011). At the conclusion of the second day of testing, participants engaged in a 12-min period of silent, eyes-closed mindfulness of breathing. The meditation began with approximately 50 s of audio instructions, recorded by Dr. Wallace:

“During the next 12 minutes, engage in the practice of mindfulness of breathing, focusing your attention on the tactile sensations at the apertures of your nostrils or just above your upper lip. With each inhalation arouse your attention and focus clearly on these tactile sensations. With each out-breath continue to maintain your attention upon the tactile sensations, while relaxing your body and mind, releasing any involuntary thoughts that may arise. So in this way maintain an ongoing flow of mindfulness, arousing with each in-breath, relaxing with each out-breath.”

A recorded chime signaled the end of the meditation period. Continuous EEG was recorded over the entire period. However, due to errors in data acquisition, only the first 6 min of data were recorded for some subjects. To allow for comparisons across all time points and groups, analyses were restricted to the first 6 min of meditation. Prior to beginning the meditation, participants were instructed to rest quietly without engaging in formal meditation with their eyes closed for 1 min while baseline EEG was recorded. These baseline data were subsequently used to calculate the IAF values for each participant at each assessment.

### Preprocessing

#### Data acquisition and filtering

EEG was acquired at a sampling rate of 2048 Hz using the BioSemi ActiveTwo system (http://www.biosemi.com) and FMS electrode caps (http://www.easycap.de) fitted with BioSemi electrode holders in an 88-channel equidistant montage. Individual electrode locations were localized in three-dimensional space using a Polhemus Patriot digitizer (http://www.polhemus.com). Due to participant discomfort (e.g., pressure on the forehead) some channels were not inserted in the cap; data from these channels were discarded. Inspection also revealed channels with poor signal quality (intermittent connectivity or extreme amplitude), which were not included in the analysis. The data were then filtered at 0.1–200 Hz (zero-phase; roll-off: 12 dB LP/24 dB HP) and referenced to the average of all remaining channels.

#### Separating neural and non-neural signal sources

Second-order blind source identification (SOBI; Belouchrani et al., 1997) was used to separate sources of contaminating signal from ongoing brain electrical activity. SOBI uses joint-diagonalization of correlation matrices at multiple temporal delays (41 delays were used, τ = [1:1:10, 12:2:20, 25:5:100, 120:20:300] ms, as described in Tang et al., 2005) to derive signal components that have a continuous time course and fixed spatial projections, referred to as *sources*. These sources can be used to generate power spectra for frequency domain analyses or as inputs for source modeling methods to estimate the probabilistic locations of the underlying neural generators. SOBI has two main advantages over other methods (e.g., ICA) for blind source identification: (a) it uses average statistics over multiple temporal delays and hence is less susceptible to outliers; and (b) it uses second-order statistics such that short segments of data are sufficient for estimating components (for further discussion of blind source separation methods see Joyce et al., 2004; Tang et al., 2005; Congedo et al., 2008; Tang, 2010).

The 6 min of continuous meditation EEG data were divided into 6 one-minute segments. This was done to facilitate future within-session longitudinal analyses beyond the scope of the present paper. Each segment was divided into non-overlapping 1-s epochs and submitted to SOBI. SOBI generated an array of maximally uncorrelated spatial sources along with their corresponding time series. For each separate participant, assessment, and 1-min epoch, the number of generated sources was equal to the number of scalp channels (~104,000 individual sources). To distinguish the neural or non-neural origin of these sources, signal components must be identified and evaluated. Although it is possible to evaluate these sources manually using quantitative features such as topography, time series, and power spectrum, the amount of data makes a manual approach infeasible. On the other hand, fully automatic solutions are harder to validate. Thus, a novel semi-automatic artifact removal tool (SMART; Saggar, 2011) was constructed to maximize the likelihood that only non-neural (i.e., artifactual) sources were rejected and that neural sources were retained.

**Figure 1:**
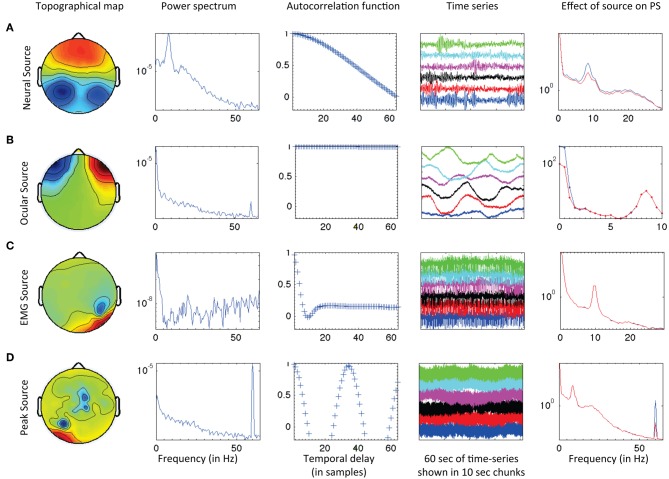
**Examples of (A) neural, (B) ocular, (C) EMG, and (D) peak sources retrieved through SOBI and classified using SMART.** SMART extracts several features from the data for initial classification of the sources and generates an html report for quick and efficient quality control. See text for details.

SMART uses a combination of features to perform an initial classification of signal component sources for inclusion or exclusion in the subsequently analyzed data. Component sources are classified according to scalp voltage topography, power spectrum, autocorrelation, time series characteristics, and the impact of each source on the overall power spectrum. SMART provides the user with an html-based interface of initial classifications. The user can quickly review all the sources and, if required, reclassify the initial classifications provided by SMART. Figure 1 illustrates several types of sources (rows) separated by SOBI, along with the features (columns) extracted by SMART. The top row **(A)** indicates a source classified as neural based on broad topography, decreasing spectral power (with a peak in the alpha-band), gradual fall-off of the lagged autocorrelation with delay, bursting pattern typical of ongoing EEG, and when the source is removed, a reduction in global power spectrum (7–15 Hz range). Row **(B)** illustrates an ocular source. This is indicated by an extreme anterior topography, lack of peaks in the power spectrum, high autocorrelation independent of lag, and a slow time series. The rightmost column shows that removal of this source affects very low frequencies of the power spectrum (<3 Hz). Row **(C)** illustrates the profile of an EMG source, with localized right posteriolateral topography, an irregular power spectrum with increased power as frequency increases, a sudden drop in autocorrelation within 10 samples indicating a high degree of noise in the time series (seen in the high frequency content of the time series), and no effect on the global power spectrum within frequencies below 30 Hz. The bottom row **(D)** illustrates a “peak” source (power line interference at 60 Hz) with localized topography indicating the affected electrode, a large peak at 60 Hz in the power spectrum, a cyclical lagged autocorrelation and uniform high frequency time series typical of a 60 Hz source. Removal of this source affects only the global power spectrum at 60 Hz.

#### Reconstruction, quality check, and conversion to standard space

Following application of SOBI and SMART, the putative non-neural sources were treated as artifacts and removed from the data. The original montage of 88-channel data was thus reconstructed using only these signal sources of presumed neural origin. The reconstructed multichannel time series was then scanned for high amplitude transient signals, or signal gaps, that may have been included in the reconstruction because they were correlated with other neural activity. For instance, large movement artifacts account for such a large percentage of the signal at the time they occur that removing the SOBI source that contains such artifacts leaves near-zero signal upon reconstruction. This post-SOBI reconstruction signal check was conducted on each reconstructed data file using the artifact scan tool in Brain Electrical Source Analysis (BESA 5.2; www.besa.de). Amplitude and gradient epoch (1 s) rejection thresholds were set individually for each participant at each assessment (gradient threshold: *M* = 4.6 μV/s, SD = 0.5; amplitude threshold: *M* = 104.4 μV, SD = 16.2).

Finally, the reconstructed 88-channel EEG data were transformed into a standard 81-channel montage (international 10–10 system) using spherical spline interpolation (λ = 2 × 10^−6^) (Perrin et al., 1989) as implemented in BESA 5.2. This transformation ensured that the number of channels was consistent across participants and that channel locations were standardized. Eight channels (AF9, Fp1, Fpz, Fp2, Nz, AF10, CB1, CB2) from the 81-channel montage were excluded because data from the corresponding nearest electrode sites were not available in the original montage, yielding a final 73-channel montage for the reconstructed EEG. These data were then transformed to a reference-free estimation of scalp current density (CSD) to limit the effects of volume conduction and improve the spatial resolution depicted on the scalp surface (e.g., Kayser and Tenke, 2006). CSD was calculated using the surface Laplacian estimated as a second derivative of the scalp potential with CSDToolbox (Kayser and Tenke, 2006; λ = 1 × 10^−6^).

### Spectral analysis

#### Power spectrum estimation

The 6 min of continuous reconstructed data were divided into 2-s (4096 point) segments with 75% overlap. Power spectra were then calculated for each of these segments using the multi-tapered power spectral density estimation method (Mitra and Pesaran, 1999; Oostenveld et al., 2011). Multi-tapered estimation reduces the bias in power spectra estimation by obtaining multiple estimates from each sample.

#### IAF and individual frequency bands

IAF was estimated using the center of gravity method for the frequency range of 7 Hz (*f*_1_)−14Hz (*f*_2_) (Klimesch, 1999),

$$\alpha_{\text{IAF}}=\frac{\sum_{i=f_{1}}^{f_{2}}\left(a(f_{i})\times f_{i}\right)}{\sum_{i=f_{1}}^{f_{2}}a(f_{i})},$$

where power-spectral estimates at *f*_*i*_ are denoted by *a*(*f*_*i*_). The α_IAF_ values were calculated for each channel and were averaged across all channels to obtain a single IAF value per subject. So as not to confound a trait measure with possible task-related effects, we calculated IAF separately during the pre-meditation baseline period and during meditation. IAF values obtained during the 1-min baseline period were used to anchor the frequency range definitions of all EEG bands. The frequency band definitions were computed separately for each participant at each assessment. Frequency ranges based on IAF are provided in Table 1, along with traditional fixed bandwidth definitions. IAF values obtained during the 6-min meditation period served as an outcome measure of possible training-related change. Across groups and retreats, IAF estimates during pre-meditation baseline and FA meditation were strongly correlated at pre- (*r* = 0.95, *p* < 0.001), mid- (*r* = 0.97, *p* < 0.001), and post-assessments (*r* = 0.96, *p* < 0.001) indicating a close correspondence of these two measures.

**Table 1:** **Comparison between fixed frequency bands and ranges based on IAF**.

	**Frequency range based on IAF**	**Frequency range (fixed)**
Delta	2–0.4 × α_IAF_ Hz	0.1–4 Hz
Theta	0.4 × α_IAF_−0.6 × α_IAF_ Hz	4–8 Hz
Alpha	0.6 × α_IAF_−1.2 × α_IAF_ Hz	8–13 Hz
Beta	1.2 × α_IAF_−30 Hz	13–30 Hz
Gamma	30–50 Hz	30–100 Hz

#### Non-parametric cluster-based permutation testing

Non-parametric cluster-based permutation testing was used to determine spatiotemporal changes in spectral band power while accounting for the problem of multiple comparisons (Maris and Oostenveld, 2007). In contrast to the traditional method of dividing scalp channels into predefined regions and calculating parametric statistics based on the average value across channels within a region, the non-parametric cluster-based approach is data driven, and may more accurately reflect the scalp topography of cortical activations. For instance, if an effect occurs in a scalp location that crosses the border between two predefined regions, the cluster approach will provide more statistical power to detect significant differences between conditions than the traditional approach.

**Figure 2:**
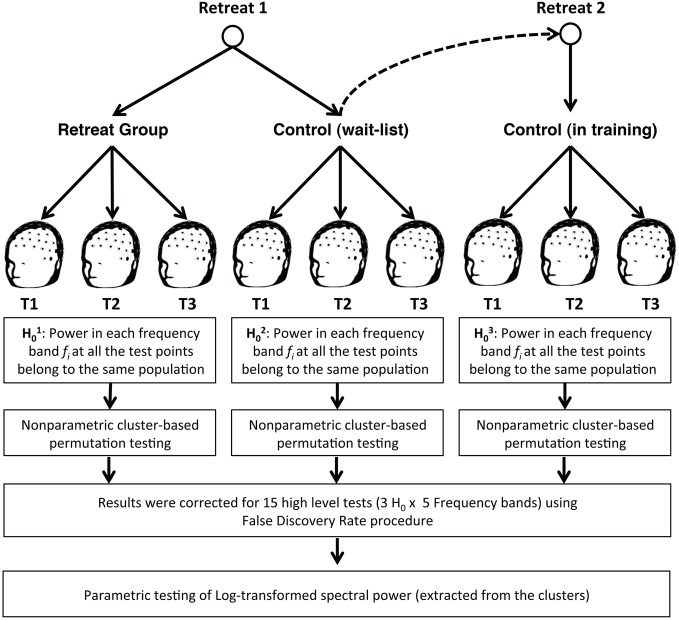
**Groups were tested three times during each three-month retreat period: at the beginning (pre-assessment), middle (mid-assessment), and end (post-assessment) of each retreat.** After estimating spectral power in each band, non-parametric cluster-based permutation analysis was utilized, followed by FDR correction for 15 high level non-parametric tests. A parametric approach was then used to examine changes in log-transformed spectral power (in clusters identified during non-parametric cluster analysis) across group and assessment.

For each frequency band, (delta, theta, alpha, beta, and gamma), retreat (Retreat 1, Retreat 2), and group (retreat, wait-list control), a separate non-parametric cluster-based permutation test (Maris and Oostenveld, 2007) was performed using FieldTrip (Oostenveld et al., 2011) to find contiguous clusters of electrodes that differed in power as a function of assessment (pre-, mid-, and post-retreat testing). The minimum cluster size was set to three electrodes, with no maximum limit. Ten thousand permutations were run to assess the significance of clusters, using a Monte Carlo estimation of significance. Significant clusters indicate changes over assessments for the respective frequency band. False discovery rate (FDR; Benjamini and Hochberg, 1995) was used to control for individual testing of each retreat, group, and frequency band (Figure 2).

#### Parametric testing of clusters

A hybrid non-parametric/parametric approach was used to assess training-related changes in spectral band power (Figure 2). After the clusters were identified and FDR-corrected for each group and assessment combination, the spectral power for each cluster was extracted by averaging the values of all identified electrodes in that cluster. These values were log-transformed to normalize their distribution and were used in parametric analyses of training-related changes in EEG spectral power.

## Results

### Spectral power

**Figure 3:**
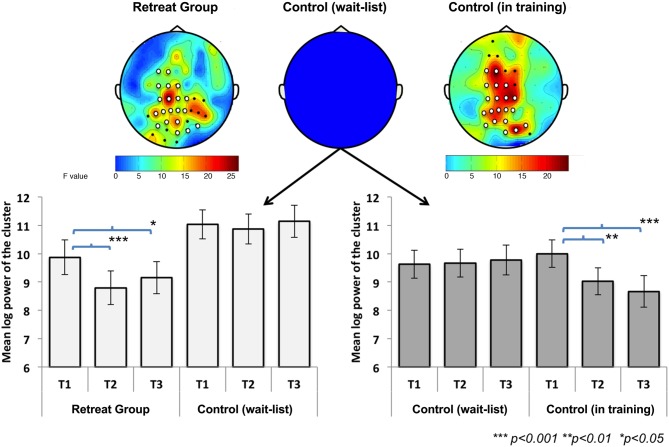
**Change in mean beta-band (1.2 × α_IAF_−30 Hz) log power [(μ V/m^2^)^2^/Hz] across assessments during Retreat 1 (left panel) and Retreat 2 (right panel).** Error bars are reported as standard errors of the mean. Significant bilateral anteriocentral and posterior clusters were found in both retreat groups. No significant clusters were found for the wait-list control group. The cluster locations for each retreat group showed substantial overlap (white electrodes). The topographic plots show the *F*-statistic result of the non-parametric test across assessments, with warmer colors (orange/red) indicating a stronger effect.

For Retreat 1, non-parametric cluster-based permutation tests revealed a significant midline-anteriocentral-posterior cluster for beta-band power (1.2 × IAF to 30 Hz) in the initial retreat group. No significant beta-band cluster was found in the control group. Additionally, no significant clusters were found for any other frequency bands for either group during Retreat 1. Separate cluster analyses for Retreat 2 also revealed a significant beta-band cluster over midline-anteriocentral-posterior regions. There was substantial overlap (20 electrodes) between beta-band clusters found for the retreat group during Retreat 1 (31 electrodes), and the wait-list controls during Retreat 2 (27 electrodes). See Figure 3 for the topographic similarity of change in spectral power for the two independent retreat groups.

Significant beta-band clusters indicate spatiotemporal changes in beta-band activity over time for participants who received training in Retreat 1 and Retreat 2, but not for the wait-list controls tested during Retreat 1. To examine the directionality of these training-related changes, multivariate repeated-measures analyses of variance (ANOVA) were used. For Retreat 1, the ANOVA included the within-subjects effect of assessment (pre-, mid-, and post-testing), the between-subjects effect of group (retreat, control), and the interaction between the two. Because no significant beta-band cluster was found for the control group in Retreat 1, data for this group consisted of the log-transformed beta-band power averaged across the same electrode locations as were found for the significant electrode cluster for the Retreat 1 group. The ANOVA revealed significant main effects of group [*F*_(1, 42)_ = 5.01, *p* = 0.031] and assessment [*F*_(2, 41)_ = 13.03, *p* < 0.001]. Importantly, a significant group × assessment interaction was also found [*F*_(2, 41)_ = 7.11, *p* < 0.01], suggesting training-related changes in beta-band power. To further explore this interaction, we conducted separate repeated-measures ANOVAs for each group with assessment as a within-subjects factor. A significant effect of assessment was found for the retreat group [*F*_(2, 20)_ = 40.12, *p* < 0.001], and *post-hoc* pairwise *t*-tests (all reported *p*-values are Bonferroni corrected for three comparisons) revealed a significant reduction in beta-band power at the mid- [*t*_(21)_ = 8.65, *p* < 0.001] and post-assessments [*t*_(21)_ = 2.72, *p* =.038]. No significant differences were found in the control group (see Figure 3).

To test the effects of training on beta-band power in Retreat 2, a repeated-measures ANOVA was used to examine the within-subjects effect of assessment (pre-, mid-, and post) in wait-list participants as they underwent training during the second retreat. Log-transformed beta-band power was averaged across the cluster found for Retreat 2 (discussed above). A repeated-measures ANOVA revealed a significant effect of assessment [*F*_(2, 20)_ = 17.20, *p* < 0.001]. *Post-hoc* tests revealed a significant reduction in beta-band power at mid- [*t*_(21)_ = 4.43, *p* < 0.001] and post-assessments [*t*_(21)_ = 5.89, *p* < 0.001], compared to the pre-assessment. Thus, the pattern and spatial topography of training-related changes in beta-band power was replicated in Retreat 2 (see Figure 3).

Retreat 1 analyses indicated no reduction in beta-band power over time in wait-list controls. Although a lack of a significant beta-band cluster in wait-list controls during Retreat 1 suggests an absence of beta-band change over time, comparisons with retreat group participants were based on the significant cluster for the Retreat 1 group only. Therefore, we conducted a follow-up analysis to further rule out the potential effects of applying the initial retreat group's cluster to assess change in the control group participants (i.e., a cluster not specific to the control participants' scalp activity). A repeated-measures ANOVA was used to test the effect of assessment on beta-band power for wait-list controls during Retreat 1 using the cluster identified for these same participants once they received training in Retreat 2. As was observed when using the initial retreat group cluster, there was no significant effect of assessment [*F*_(2, 20)_ = 0.18, *p* = 0.84], suggesting that the lack of beta-band reduction in wait-list controls in Retreat 1 was not dependent on using the Retreat 1 training group-defined electrode cluster.

Taken together these analyses suggest that intensive FA training is associated with a reduction in beta-band power during mindfulness of breathing, with effects most reliably observed bilaterally overlying medial prefrontal, central, and parietal brain regions.

### Individual alpha frequency

**Figure 4:**
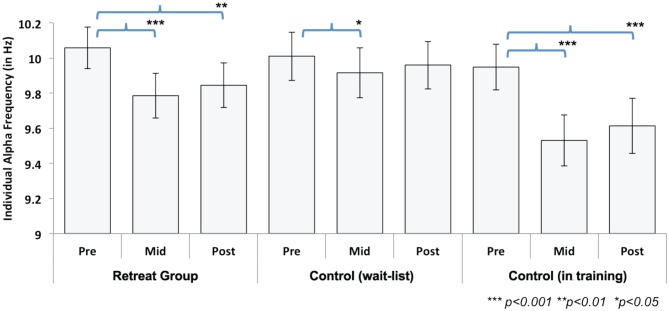
**Change in individual alpha frequency (IAF) across assessments during Retreat 1 (Retreat and Control group) and Retreat 2 (Control group in training).** The figure shows IAF values, averaged across participants, in each group and at each assessment. Error bars are reported as standard errors of the mean (SEM). Significant training-related reductions in IAF were found in both retreat groups at mid- and post-assessments (compared to the pre-assessment).

IAF values were calculated during the 6 min of FA meditation at each assessment point for the separate retreats. Changes in IAF during Retreat 1 were examined using multivariate ANOVA in a manner analogous to the beta-band power analyses summarized above. A 3 (assessment) × 2 (group) ANOVA revealed a significant main effect of assessment [*F*_(2, 41)_ = 23.26, *p* < 0.001], indicating that IAF values shifted across time. The main effect of group [*F*_(1, 42)_ = 0.13, *p* = 0.72] was not significant. As predicted, a significant group × assessment [*F*_(2, 41)_ = 6.40, *p* < 0.01] interaction was found, suggesting training-related shift in IAF across three months of meditation training. To further explore this interaction, we conducted separate repeated-measures ANOVAs for each group. There was a significant main effect of assessment for both the initial retreat [*F*_(2, 20)_ = 20.89, *p* < 0.001] and the wait-list control [*F*_(2, 20)_ = 5.38, *p* = 0.014] groups. For the retreat group, Bonferroni-corrected follow-up comparisons revealed that IAF decreased at mid- [*t*_(21)_ = 6.59, *p* < 0.001] and post-assessments [*t*_(21)_ = 4.50, *p* < 0.001], compared to the pre-assessment. In the wait-list control group, IAF also decreased at the mid-assessment [*t*_(21)_ = 2.61, *p* = 0.049] as compared to the pre-assessment. Neither group showed significant changes in IAF between mid- and post-assessments (Figure 4).

In order to test changes in IAF during Retreat 2, a repeated-measures ANOVA was used to examine the within-subjects effect of assessment. A significant main effect was found [*F*_(2, 20)_ = 35.44, *p* < 0.001]. Similar to the observed pattern for the initial retreat group during Retreat 1, a decrease in IAF was found at both the mid- [*t*_(21)_ = 7.30, *p* < 0.001] and post-assessments [*t*_(21)_ = 6.00, *p* <0.001], as compared to the pre-assessment (Figure 4). Again, no reductions in IAF were found between the mid- and post-assessments.

The overall pattern of results suggests training-related decreases in IAF in both retreats. Although these data demonstrate reliable training-related reductions in IAF across three months of meditation training, reductions in IAF were also observed between pre- and mid-assessments for the wait-list controls in Retreat 1. However, the effect size for pre-to-mid change in IAF was nearly three times as large for the training groups in both Retreat 1 (*Cohen*'*s d* = 1.40) and Retreat 2 (*d* = 1.56) than in wait-list controls (*d* = 0.56).

### The effect of daily meditation on beta-band power and IAF

Hierarchical multiple regression analysis was used to examine whether decreases in beta-band power and/or shifts in IAF were related to the amount of average self-reported daily FA meditation. Because the pattern of training-related change in beta and IAF was similar across both retreats, data from both groups were combined.

In the first step of the regression model of beta change, pre-assessment beta was entered as a predictor of post-assessment beta in order to account for baseline beta-band power prior to training. As expected, pre-assessment beta significantly predicted post-assessment beta [*R*^2^ = 0.804, *F*_(1, 42)_ = 171.79, *p* < 0.001]. The second step included the average daily amount of FA mediation practiced by each participant in order to examine the unique variance explained by daily practice in post-assessment beta independent of pre-assessment beta. In step 2, the addition of average daily FA hours did not add significantly to the explained variance of the model [Δ*R*^2^ = 0.003, Δ*F*_(1, 41)_ = 0.57, *p* = 0.46]. Thus, collapsed across retreats, changes in beta were not predicted by the amount of participants' daily FA meditation practice.

In a similar step-wise manner, we used a hierarchical regression model to examine whether the amount of FA meditation predicted changes in IAF. In the first step, pre-assessment IAF significantly predicted post-assessment IAF [*R*^2^ = 0.870, *F*_(1, 42)_ = 280.134, *p* < 0.001; see Table 2]. In step 2, the addition of average daily FA meditation hours added significantly to the explained variance of the model [Δ*R*^2^ = 0.020, Δ*F*_(1, 41)_ = 7.45, *p* = 0.009]. This relation was negative (β = −0.142), indicating that the more the participants engaged in FA meditation the more IAF decreased. In contrast to changes in beta-band power, these results suggest that reductions in IAF are significantly predicted by the amount of daily FA meditation engaged in over the course of meditation training.

**Table 2:** **Predicting changes in IAF from average daily focused attention meditation**.

	***B***	**SE**	β	***t*-statistic**	***p***
**Step 1 (*R*^2^ = 0.870)**					
Constant	−1.117	0.649	–	−1.721	0.093
IAF at pre-assessment	1.084	0.065	0.933	16.737	<0.001
**Step 2 (*R*^2^ = 0.890)**					
Step 1 predictor repeated	–	–	–	–	–
Average daily FAM	−0.065	0.024	−0.142	−2.729	0.009

Note: The dependent variable in each regression is IAF at post-assessment. Data from RG1 and RG2 were combined for these analyses (N = 44).

### The effect of altitude on beta-band power and IAF

During Retreat 1, the retreat group lived onsite at the retreat center for the full three-month training period, whereas wait-list control participants lived at home and were flown to the retreat center at each assessment point for testing. Because the retreat center was located at a relatively high altitude (~2500 m), it is possible that traveling to and living at that higher altitude could have influenced changes in spectral power and/or IAF over time (Kaufman et al., 1993; Guger et al., 2005). To rule out this explanation, we used hierarchical multiple regression analysis to examine whether the reductions in beta-band power and IAF could be predicted by the difference in elevation between the testing location and the participant's city of residence. All participants except one resided at a lower elevation than the retreat center (*N* = 43; *M*_*elevation_difference*_ = 1770.12 m, *SD*_*elevation_difference*_ = 767.21 m); the single participant who resided at a slightly higher elevation than the retreat center was excluded from analysis. As in the analysis of daily FA meditation, the first step of each regression model included the pre-assessment level of beta or IAF, respectively. The addition of altitude change in step 2 did not add significantly to the explained variance of the model for either beta-band power [Δ*R*^2^ = 0.01, Δ*F*_(1, 41)_ = 2.26, *p* = 0.14] or IAF [Δ*R*^2^ = 0.003, Δ*F*_(1, 41)_ = 0.85, *p* = 0.36]. These analyses suggest that changes in IAF and beta-band power were unrelated to changes in altitude.

## Discussion

We observed training-related changes in oscillatory cortical activity in a longitudinal, wait-list controlled study of three-months of intensive meditation training. Spectral analysis of dense-array scalp-recorded EEG was used to characterize training-related changes during 6 min of FA meditation. Second-order blind source identification and a novel semi-automatic artifact removal tool were utilized to identify and remove non-neural signal contaminants. Each participant's resting-state IAF was estimated and used to define frequency bands. Non-parametric cluster-based analysis revealed consistent training-induced changes in the beta-band only. In an initial retreat, significant reductions in beta-band power were observed bilaterally in anterior-central and posterior scalp regions with training. This pattern was replicated in a second retreat, in which the wait-list control group received formally identical training. A reduction (slowing in overall alpha frequency) was also observed in state-related IAF across both retreats. Moreover, the degree of reduction in IAF was predicted by the amount of time participants practiced FA meditation during training. The robustness of these findings, replicated across separate training periods, provide evidence of specific longitudinal changes in characteristic brain oscillatory activity obtained during mindfulness of breathing.

### Reductions in beta-band power

Previous research has demonstrated that beta-band power is inversely related to cortical activity and excitability (Tamura et al., 2005; Ploner et al., 2006), and that beta-band power over somatosensory cortex is inversely correlated with fMRI-dependent BOLD activation (Ritter et al., 2009). Suppression of ongoing oscillatory activity in the beta and alpha bands may result from increased cellular excitability in thalamocortical networks (Steriade, 2006; Bollimunta et al., 2011) and may serve to facilitate selective sensory gating, augmenting the signal-to-noise ratio of incoming sensory information (Pfurtscheller and Lopes da Silva, 1999; van Ede et al., 2011). This suggests a functional role for beta-band activity within tasks involving attention to tactile stimuli. Specifically, anticipatory modulations in beta-band power have been associated with spatiotemporal orienting of attention (Dockstader et al., 2010; Jones et al., 2010; van Ede et al., 2010, 2011) and conscious detection of subtle tactile stimuli (Linkenkaer-Hansen et al., 2004; Schubert et al., 2009). For example, when attention is cued to a lateralized tactile stimulus, beta-band power over parietal cortex is suppressed contralateral and increased ipsilateral to the attended stimulus (van Ede et al., 2011). The degree of prestimulus suppression is associated with both faster responding and enhanced stimulus detection (Linkenkaer-Hansen et al., 2004; van Ede et al., 2011).

Suppression of beta-band power may therefore reflect increased cortical activity associated with enhanced sensory processing of ongoing tactile stimuli. Training-related suppression of beta-band power in the present study is in line with the functional role proposed for oscillatory activity in facilitating sensory processing of the attended breath stimulus. During each assessment, participants focused their attention on the dynamic changes in breath sensations over 6 min of eyes-closed meditation. They were instructed to notice subtle tactile sensations at the aperture of the nostrils while regulating attention should it lapse. Thus, reductions in beta-band power over the course of training may reflect increased cortical activation of sensory-related attentional networks and an increased capacity to focus attention on the breath during mindfulness of breathing meditation. Beta suppression may also reflect increased perceptual discrimination and conscious perception of these tactile sensations. In support of this idea, Schubert et al. (2009) found that the absolute magnitude of beta suppression across individuals was associated with a greater ability to perceive target tactile stimuli within a context of similar distracters. In the present cohort, training was previously reported to increase perceptual discrimination of subtle visual stimuli (MacLean et al., 2010). In a similar manner, we speculate that intensive meditative practice may result in increased levels of sensory processing of ongoing tactile stimuli.

The observed reduction in beta-band power following meditative training is also consistent with a cross-sectional study of highly experienced meditative adepts (Brefczynski-Lewis et al., 2007). Brefczynski-Lewis et al. (2007) observed increased BOLD activation in brain regions typically involved in sustained attention during FA meditation for expert meditators with an average of 19,000 h of practice. In contrast, experts with over 40,000 h of lifetime practice showed a decreased amount of activation in the same brain regions during FA meditation. These results suggest that cortical activation during meditative practice may follow a curvilinear trajectory such that both novices and highly experienced practitioners show less attention-related activation than practitioners whose lifetime experience falls between these extremes. The average level of lifetime meditation experience among participants in our study was about 2500 h—a moderate level in comparison to the above groups. Thus, our observed reductions in beta-band power, presumably indicative of an increase in cortical activation, are in line with the trajectory of training-related change proposed by Brefczynski-Lewis et al. (2007).

Finally, the observed pattern of training-related modulation of oscillatory activity may be associated with improvements in behavioral measures of attentional performance. In this same cohort, we previously reported improvements in sustained attention, response inhibition, and perceptual discrimination following training (MacLean et al., 2010; Sahdra et al., 2011). Repeated engagement of attention networks during practice may allow for more efficient resource allocation during demanding external psychophysical tasks. For example, changes in neural signatures of attentional stability have been found following three months of intensive Vipassana meditation, which includes FA meditation as one component of training (Lutz et al., 2009). This link should be examined in future work by directly relating measures of cortical activation during practice and subsequent training-related behavioral outcomes. Furthermore, theoretical approaches, such as computational modeling, can be employed to test targeted hypotheses regarding the underlying cortical dynamics involved in these processes.

### Reductions in individual alpha frequency

We also observed training-related reductions in IAF during FA meditation, which were evident by the midpoint of each retreat. IAF has typically been conceptualized as a relatively stable index of an individual's trait-like capacity for cognitive resource allocation (Kondacs and Szabó, 1999; Jann et al., 2010) and cognitive load (Moran et al., 2010). Although the available literature on IAF provides little framework for conceptualizing intraindividual change as a result of training, we believe that diminished IAF may represent an overall reduction in cognitive effort during meditation. Peak alpha frequency has been shown to increase with elevated cognitive load in visuospatial working memory (Moran et al., 2010) and may reflect a greater allocation of resources to the maintenance of information in memory. After intensive training, increased attentional stability may reduce the attentional resources required to sustain attention on the sensations of the breath. Additionally, meditation training may promote greater efficiency in the reorienting of attention to a given stimulus. Although the aforementioned reductions in beta-band power suggest enhanced sensory processing of the tactile sensations of the breath, we speculate that the observed reductions in IAF may indicate that such increases in activity incur fewer global processing costs.

A reduction in IAF was observed in both the training and control groups in the first half of the first retreat, suggesting that these changes in IAF were due at least in part to task-related learning. By the second assessment, both the retreat and the wait-list control groups had previous exposure to the demanding testing procedures (i.e., the perceptual and cognitive tasks that occurred in the 2–3 h before the FA meditation period), and thus reductions in IAF during FA meditation may have been due in part to increased familiarity with and reduced stress caused by data collection procedures. However, the reduction in IAF in controls was of a moderate effect size, whereas the effect size in the training groups was nearly three times larger. In addition, the amount of time participants devoted to FA meditation over the course of three months significantly predicted changes in IAF from pre- to post-retreat training assessments. This evidence suggests that a substantial degree of the change in IAF observed in retreat groups was related to meditation training.

### Contributions and limitations

Contrary to our predictions, we did not find reductions in alpha-band power following training. Although reductions in power in the alpha-band have been associated with anticipatory attention to tactile stimuli in both training (Kerr et al., 2011) and non-training (Jones et al., 2010; van Ede et al., 2011) contexts, alpha has been primarily implicated in the visuospatial domain (Thut et al., 2006; van Dijk et al., 2008; Romei et al., 2010). In particular, increased alpha-band activity may serve as a mechanism by which visual selective attention acts to suppress distracting information (Foxe and Snyder, 2011). Thus, it is reasonable to expect that training-related changes in alpha-band power might be more likely to manifest during focused meditation on a visual object (e.g., Brefczynski-Lewis et al., 2007) and/or during behavioral performance of visual attention tasks. In addition, it is interesting to note that our findings do not support a pattern of increased alpha power during meditative states, as has been reported in a number of previous studies (Cahn and Polich, 2006). As alpha may reflect non-specific effects such as general arousal level, we believe this highlights the importance of testing targeted hypotheses concerning the process-specific modulation of task-specific (e.g., tactile processing during mindfulness of breathing) cortical activity during meditation.

Previous studies have reported increases in gamma-band activity during meditation in experienced practitioners (Lutz et al., 2004; Cahn et al., 2010). Analysis of gamma-band power in humans is notoriously challenging due to the contribution of non-neural sources. Scalp-recorded muscle activity generates broadband myogenic electrical “noise” that overlaps substantially with the gamma band, which may also influence alpha- and beta-bands (McMenamin et al., 2010, 2011). In the present study, we utilized novel signal processing methods to remove such putatively non-neural signal sources from the ongoing EEG. This may have contributed to the lack of changes in gamma-band activity with training. Furthermore, in contrast to previous studies that used standard ranges for each frequency band, we defined the range of each spectral band according to each participant's IAF during rest. This approach accounts for individual differences in the frequency band boundaries and therefore may provide a more accurate measure of activity. For example, the beta-band began at ~11.6 Hz in the current study, which is lower than the traditional lower boundary of 13 Hz. In the present study, activity in the IAF-defined frequency bands may have cut across the traditional boundaries of fixed spectral bands. Thus, for the majority of participants in our study, the IAF-defined beta-band likely included activity from both the traditionally-defined alpha (~8–13 Hz) and beta (~13–30 Hz) bands.

Certain factors unrelated to the training may have contributed to the present findings. First, the retreats took place in a remote mountain setting at an altitude of approximately 2500 m above sea level, where participants spent most of their time in silent, solitary meditation. Although change in altitude is known to affect EEG recordings, typically by increasing the amplitude of the signal (Kaufman et al., 1993), no statistical relation was found between altitude and either beta-band or IAF change. Furthermore, it is possible that exposure to the natural wilderness setting may have cognitive benefits independent of the training (e.g., Berman et al., 2008). Second, beta-band power has been implicated in the active maintenance of a current motor set (Engel and Fries, 2010) such that beta-band activity increases in preparation of an anticipated postural challenge (Androulidakis et al., 2007). In the present study, training consisted of maintaining meditative posture for lengthy periods of time. Future research should therefore explore the potential effects of posture on state-related meditation effects. Finally, motivational levels may not have been exactly matched across groups. Participants receiving training were not blind to their group assignment and therefore our results may have been susceptible to demand characteristics resulting from varying levels of influence from teacher expectations and commitment to a general Buddhist worldview. Although our wait-list design likely addressed several important design limitation of prior research, future investigators should attempt to account for social, motivational, and environmental factors by implementing active comparison conditions in which participants complete non-meditative training or activities in a retreat-like setting.

In summary, we found replicable and robust reductions in beta-band power over central-parietal regions and decreased IAF following three months of intensive FA meditation. These findings add to the growing body of literature demonstrating functional brain changes associated with meditation practice—changes that may underlie generalized improvements in cognition and psychological well-being.

### Conflict of interest statement

The authors declare that the research was conducted in the absence of any commercial or financial relationships that could be construed as a potential conflict of interest.
